# Assessing the impact of annual health screenings in identifying noncommunicable disease risk factors within Qatar’s primary health care corporation Qatari registered population

**DOI:** 10.3389/fpubh.2024.1305636

**Published:** 2024-05-23

**Authors:** Samya Ahmad Al-Abdulla, Ahmad Haj Bakri, Mariama Aminata Mansaray, Mohamed Ghaith Al-Kuwari

**Affiliations:** ^1^Primary Health Care Corporation (PHCC), Doha, Qatar; ^2^College of Medicine, Qatar University, Doha, Qatar

**Keywords:** noncommunicable diseases, annual health screening, risk factors, prevention, Qataris

## Abstract

**Background:**

Noncommunicable diseases (NCDs) are a significant global health burden, including in Qatar, where cardiovascular diseases cause mortality. This study examines the outcomes of the annual health checkup implemented by the Primary Health Care Corporation (PHCC) in Qatar in detecting NCDs risk factors among Qataris aged 18+ years.

**Methods:**

A cross-sectional study design was implemented to calculate the prevalence of behavioural and metabolic NCDs risk factors among Qataris who underwent annual health checkups between 2017 and 2019. Data on age, gender, tobacco consumption, height, weight, blood pressure, glycated haemoglobin (HbA1c), and cholesterol levels were extracted from electronic medical records.

**Results:**

In 2019, Qatar experienced an 80% rise in Annual Health checkups attendance compared to 2017. Tobacco use fluctuated between 11.79 and 12.91%, peaking at 35.67% among males in 2018. Qataris with elevated blood pressure dropped from 29.44% in 2017 to 18.52% in 2019. Obesity decreased from 48.32 to 42.29%, more prevalent in females. High HbA1c levels reduced from 13.33 to 8.52%, while pre-diabetic levels rose from 21.1 to 25.52%. High cholesterol ranged from 7.31 to 9.47%. In a regression analysis, males had 2.28 times higher odds of elevated blood pressure and 1.54 times higher odds of high HbA1c, with a 0.68 lower odds of obesity compared to females. Ages 36 and above had 2.61 times higher odds of high cholesterol compared to younger age groups.

**Conclusion:**

The annual health screening has shown promising results in detecting and addressing NCDs risk factors among Qataris. The attendance rate has increased over the three-year period, and there has been a decrease in the prevalence of elevated blood pressure, obesity, and high HbA1c levels. However, tobacco consumption and pre-diabetic levels remain significant concerns. These findings can guide the implementation of tailored preventative and curative services to improve the health and well-being of the Qatari population.

## Introduction

Globally, Noncommunicable diseases (NCDs) kill 40 million people each year (1). Cardiovascular diseases are a significant cause of health problems and deaths worldwide (2). Mortality attributed to circulatory system diseases is the highest cause of mortality among people in Qatar (3). In Qatar, the prevalence of diabetes among the Qatari population above 18 years old was 16.7%, and the prevalence of high blood pressure among the same group was 33% (4). Modifiable behavioural risk factors such as tobacco use, unhealthy diet, and physical inactivity, and harmful use of alcohol increases the risk of NCDs (5). According to STEPwise 2012, obesity in Qatar was reported at 40% among Qataris aged 18 years and over (4). The overall smoking prevalence reported in the global adult tobacco survey conducted in Qatar in 2013 was 17.9% among males (6).

The recent demographic and epidemiological trends indicate that populations tend to live longer and with higher disease burdens worldwide (7). In Qatar, the Second National Development Strategy (2017–2022) was expected to focus on healthcare, including delivering its newest National Health Strategy 2018–2022, which strongly focuses on primary care as the gateway to all other healthcare services (8). The nationally led programs to establish integrated care across the health sector will make significant improvements in ensuring patients with multiple NCDs will experience greater coordination of care and better patient outcomes (8).

Qatar has a dynamic population. In 2019, the total population was 2,666,938 inhabitants (9). The diversity of the Qatar expatriate population might pose challenges for health care services planning. Nevertheless, much of Qatar’s strategic focus is on the Qatari population, as the long-term residents of the country, and where the most significant impact on health spending and future planning can be made and measured.

The Primary Health Care Corporation (PHCC) is the significant public sector provider of primary care services to families in Qatar. PHCC operated 27 health centres in March 2020 in three main health regions – northern, central, and western. The Primary Health Care Corporation identified the regions operationally to ensure easy allocation and management of health centres. The registered active population in these health centres was 1,461,987 by February 2020 – a 55% increase since 2015 (10).

The PHCC has invested in preventive health services to enhance healthier lives for its target population with comprehensive health promotion and preventive services provided with a focus on screening, healthy lifestyle promotion, and immunisation (11). Thus, following the launch of the National Primary Health Care Strategy in 2013, the PHCC has been committed to the successful implementation of numerous pledges and recommendations found in the strategy (12). The third pledge of the strategy stipulated that primary care providers will introduce a yearly health check for the identified target population that will benefit from the check. The health check will focus on issues such as diet, exercise, smoking status, and indicators such as blood pressure (12). The latter led to the development of the PHCC annual health check services called SMART at that time, currently rebranded as Annual Health Checkup services. Since the burden of NCDs and their subsequent risk factors was high in Qatar (4). The annual health check focus has been on identifying common risk factors for ill health-related non-communicable diseases to support identifying individuals that could benefit from PHCC’s preventative care services, such as the wellness service.

Hence, the annual health checkup services were rolled out as of January 2017 across the PHCC health centres targeting Qataris aged 18 years old and over, excluding pregnant women and women attending post-natal clinics with the vision of becoming an impactful primary preventative service in Qatar and to guide patients to utilise efficiently the full range of services offered by PHCC to maintain and improve their health and wellbeing.

As such, this research aims to examine the annual health checkup outcomes in detecting non-communicable diseases and behavioural and metabolic risk factors among the Primary Health care registered Qataris aged 18 years old and over between the 1st of January 2017 and the 31st of Dec 2019 so that tailored preventative/curative services can be enhanced and implemented.

## Methods

A cross-sectional study design was applied to calculate the prevalence of behavioural and metabolic noncommunicable diseases (NCDs) risk factors among Qataris aged 18 years old and over who underwent the annual health checkup between 1 January 2017 and 31 Dec 2019. The analysis and the results were reported year by year throughout the study.

Our study included all individuals of Qatari nationality who were 18 years of age or older and were registered at the Primary Health Care Centers. Specifically, we included those invited to participate in the annual health checkup and attended their scheduled annual health assessment appointments during the specified time frame. Throughout the study period, a total of 12,620, 19,772, and 21,481 Qatari individuals aged 18 years and above were invited to undergo annual health checkups in the years 2017, 2018, and 2019, respectively. Pregnant women and women attending post-natal clinics were excluded from the analysis per the annual health check inclusion criteria for service provision.

The annual health checkup services are provided across two visits.

- Visit 1: The “Annual Health Check Assessment Appointment” with a Nurse (30 min)
- Visit 2: The “Annual health Check Consultation Appointment” with a Physician (15 min)

At the assessment appointment, the nurse takes the client’s vital signs, sits with them to complete the Health Assessment Questionnaire (Personal, Family, Social History, Mental Health, and Cancer Screening Assessment), places proposal laboratory orders, and then directs the client to the laboratory to provide blood, stool, and urine samples.

At the follow-up of the annual health Consultation Appointment, the physician reviews the information captured in the health check assessment and the laboratory test results. Based on the Physician’s clinical judgment, the client receives any necessary counselling and advice, referrals, further investigations, follow-up appointments, prescriptions, and health education materials.

As such, Data on age, gender, tobacco consumption, height, weight, blood pressure, glycated haemoglobin (HbA1c), and cholesterol reading was extracted electronically from the patient’s electronic medical records for all the persons who underwent annual health checkup visit 1 (check assessment appointment) in the aforementioned time interval via the health intelligence department at the Primary Health Care Corporation in Qatar. The accuracy of the data and the missing variables were checked by the latter department, ensuring the data confidentiality aspect.

All the Qataris aged 18 years old and over who underwent the annual health checkup-the assessment appointments were included in our analysis as per attendance year between 2017 and 2019. Therefore, no minimum sample was required because we were including all the attendees in our study.

Statistical weighting was applied to adjust for the difference in sample sizes between the years 2017, 2018, and 2019. Hence, the imperative probability weights were produced for the analysis with the following figures: 4.3, 2.3, and 2.5 to adjust for the difference in sample sizes for 2017, 2018, and 2019, respectively. Additionally, the findings were stratified by years to allow for comparing results within comparable groups and mitigate the impact of sample size variations.

To ascertain the significance of the disparities in the results of the health indicators observed yearly, statistical tests were applied, such as the chi-square test for categorical data and ANOVA for continuous data. The later tests were performed utilising STATA 14, and the subsequent *p*-values were produced and reported at the end of each table to determine whether the differences among years were statistically significant and occurred due to sampling variability/random fluctuations.

Descriptive analyses and statistical tests using STATA-14 were employed to determine the prevalence of non-communicable disease risk factors in the target population. The findings were reported in absolute numbers and percentages across different conditions, categorised by the three years of implementation and their corresponding *p*-values related to each year.

A multiple logistic regression analysis was conducted to investigate the impact of gender and age group, categorized into two groups (18–35 years and 36+ years), on individuals with a BMI of 30+ kg/m^2^, those with Glycated Hemoglobin (HbA1c) levels of 6.5% or higher, individuals with high cholesterol levels (Cholesterol >6.2 mmol/L), and those with elevated blood pressure (130/85 mm Hg or higher). The logistic regression model was developed using STATA software, accounting for variations across years.

## Results

**Figure 1:**
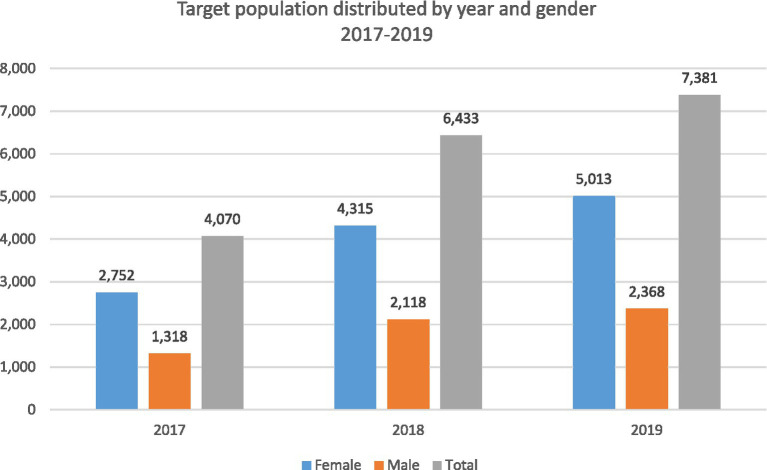
Target population distribution by gender and year 2017–2019.

A total of 4,070, 6,433, and 7,381 Qataris aged 18+ completed the annual health checkup assessment appointment in 2017, 2018, and 2019, respectively. The female-to-male ratio among those who attended the services was almost three females to 1 male across the three years of implementation. The target population who attended the annual health checkup assessment visits increased by 80% in 2019 compared to the year of inception in 2017, as indicated in Figure 1.

A total of 2,688 (909 male, 1779 female), 4,370 (1,587 male, 2,783 female), and 6,283 (2025 male, 4,258 female) Qataris had information related to their smoking behaviours in 2017, 2018, and 2019, respectively. The smoking consumption rate among both sexes ranged between 11.79 and 12.91%. The highest rate observed among males was in 2018 at 35.67%, as indicated in Table 1.

**Table 1:** Tobacco consumption rate among the target population by gender and year.

Current tobacco consumption rate by year	Both gender		Female		Male		*p*-value
	*n*	%	*n*	%	*n*	%	
2017	347	12.91	29	1.63	318	34.98	0.007
2018	609	13.93	43	1.55	566	35.67	0.007
2019	741	11.79	49	1.15	692	34.17	0.007

In 2017, 2018, and 2019, there were 4,066 (1,314 male, 2,752 female), 6,072 (1993 male, 4,079 female), and 7,192 (2,299 male, 4,893 female) Qataris with complete blood pressure measurements, respectively.

As reported in Table 2, the proportion of Qataris with elevated blood pressure—130/85 mm Hg or higher—declined throughout the three years of the annual health checkup implementation, from 29.44% in 2017 to 18.52% in 2019 among both genders.

**Table 2:** Elevated blood pressure – 130/85 mm Hg or higher among the target population by gender and year.

Elevated blood pressure – 130/85 mm Hg or higher by year	Both sexes		Female		Male		*p*-value
	*n*	%	*n*	%	*n*	%	
2017	1,197	29.44	650	23.62	547	41.63	0.000
2018	1,345	22.15	700	17.16	645	32.36	0.000
2019	1,332	18.52	651	13.30	681	29.62	0.000

The overall prevalence of obesity among Qataris who underwent the annual health assessment appointment demonstrated a decline from 48.32% in 2017 to 42.29% in 2019. However, the prevalence of obesity remained higher among Qatari females than among Qatari males, as demonstrated in Table 3.

**Table 3:** Body mass index (BMI) classifications among the target population by gender and year.

BMI Category	Female		Male		Total		*p*-value
	*n*	%	*n*	%	*n*	%	
**2017**							
Underweight – BMI <18.5 kg/m^2^	70	2.57	18	1.41	88	2.20	0.000
Normal weight – BMI 18.5–24.99 kg/m^2^	462	16.99	230	18.05	692	17.33	0.000
Overweight – BMI between 25–30 kg/m^2^	772	28.38	512	40.19	1,284	32.15	0.000
Obese – BMI 30+ kg/m^2^	1,416	52.06	514	40.35	1,930	48.32	0.000
**2018**							
Underweight – BMI <18.5 kg/m^2^	101	2.66	28	1.55	129	2.30	0.000
Normal weight – BMI 18.5–24.99 kg/m^2^	775	20.4	382	21.18	1,157	20.65	0.000
Overweight – BMI between 25–30 kg/m^2^	1,216	32.01	716	39.69	1,932	34.48	0.000
Obese – BMI 30+ kg/m^2^	1,707	44.93	678	37.58	2,385	42.57	0.000
**2019**							
Underweight – BMI <18.5 kg/m^2^	108	2.75	39	2.08	147	2.53	0.000
Normal weight – BMI 18.5–24.99 kg/m^2^	859	21.85	401	21.36	1,260	21.69	0.000
Overweight – BMI between 25–30 kg/m^2^	1,204	30.63	703	37.45	1,907	32.83	0.000
Obese – BMI 30+ kg/m^2^	1,760	44.77	734	39.10	2,494	42.94	0.000

Between 2017 and 2019, the proportion of Qataris with glycated haemoglobin (HbA1c)—6.5% or higher decreased from 13.33% in 2017 to 8.52% in 2019. The proportion of Qataris with HbA1c between 5.7 and 6.4%, marking the pre-diabetic group, demonstrated an increase from 21.1 to 25.52% in 2017 and 2019, respectively, as indicated in Table 4.

**Table 4:** Glycated haemoglobin (HbA1c) classifications among the target population by year and gender.

HBA1c category	Female		Male		Total		*p*-value
	*n*	%	*n*	%	*n*	%	
**2017**							
Glycated haemoglobin (HbA1c) – 5.6% or less	1,870	69.23	746	57.87	2,616	65.56	0.000
Glycated haemoglobin (HbA1c) – 5.7-6.4%	517	19.14	325	25.21	842	21.1	0.000
Glycated haemoglobin (HbA1c) – 6.5% or higher	314	11.63	218	16.91	532	13.33	0.000
**2018**							
Glycated haemoglobin (HbA1c) – 5.6% or less	2,738	67.82	1,100	56.24	3,838	64.04	0.000
Glycated haemoglobin (HbA1c) – 5.7-6.4%	903	22.37	582	29.75	1,485	24.78	0.000
Glycated haemoglobin (HbA1c) – 6.5% or higher	396	9.81	274	14.01	670	11.18	0.000
**2019**							
Glycated haemoglobin (HbA1c) – 5.6% or less	2,963	68.43	1,232	60.69	4,195	65.96	0.000
Glycated haemoglobin (HbA1c) – 5.7-6.4%	1,080	24.94	543	26.75	1,623	25.52	0.000
Glycated haemoglobin (HbA1c) – 6.5% or higher	287	6.63	255	12.56	542	8.52	0.000

Among Qataris who attended the annual health checkup assessment appointment, a total of 3,983 (1,282 male, 2,701 female), 5,947 (1943 male, 4,004 female), and 6,304 (2009 male, 4,295 female) had conducted lipid panel tests in 2017, 2018, and 2019, respectively. The proportion of High cholesterol level – Cholesterol >6.2 mmol/L among Qataris ranged between 9.47 and 7.31%, with males having the highest proportion at 12.17% in 2017, as shown in Table 5.

**Table 5:** Lipid profile classifications among the target population by year and gender.

	Female		Male		Total		*p*-value
	*n*	%	*n*	%	*n*	%	
**2017**							
Optimal cholesterol level – cholesterol <5.2 mmol/L	1773	65.64	758	59.13	2,531	63.55	0.000
Intermediate cholesterol level – cholesterol 5.2–6.2 mmol/L	707	26.18	368	28.71	1,075	26.99	0.000
High cholesterol level – cholesterol >6.2 mmol/L	221	8.18	156	12.17	377	9.47	0.000
High triglyceride level – 1.7 mmol/L or higher	585	21.67	515	40.17	1,100	27.63	0.000
High LDL level – LDL >4.11 mmol/L	169	6.26	149	11.63	318	7.99	0.000
Reduced high-density lipoprotein (HDL) cholesterol – less than 1.04 mmol/L in men or less than 1.3 mmol/L in women	995	36.84	409	31.90	1,404	35.25	0.000
**2018**							
Optimal cholesterol level – cholesterol <5.2 mmol/L	2,836	70.83	1,231	63.36	4,067	68.39	0.000
Intermediate cholesterol level – cholesterol 5.2–6.2 mmol/L	930	23.23	515	26.51	1,445	24.3	0.000
High cholesterol level – cholesterol >6.2 mmol/L	238	5.94	197	10.14	435	7.31	0.000
High triglyceride level – 1.7 mmol/L or higher	735	18.36	727	37.4	1,462	24.58	0.000
High LDL level – LDL >4.11 mmol/L	183	4.6	198	10.4	381	6.48	0.000
Reduced high-density lipoprotein (HDL) cholesterol – less than 1.04 mmol/L in men or less than 1.3 mmol/L in women	1,541	38.62	629	32.51	2,170	36.62	0.000
**2019**							
Optimal cholesterol level – cholesterol <5.2 mmol/L	2,957	68.85	1,232	61.32	4,189	66.45	0.000
Intermediate cholesterol level – cholesterol 5.2–6.2 mmol/L	1,035	24.1	554	27.58	1,589	25.21	0.000
High cholesterol level – cholesterol >6.2 mmol/L	303	7.05	223	11.1	526	8.34	0.000
Normal high triglyceride level- <1.7 mmol/L	689	16.05	645	32.11	1,334	21.17	0.000
High LDL level – LDL >4.11 mmol/L	246	5.78	214	10.9	460	7.39	0.000
Reduced high-density lipoprotein (HDL) cholesterol – less than 1.04 mmol/L in men or less than 1.3 mmol/L in women	1,511	35.44	543	27.12	2054	32.78	0.000

In a multivariable logistic regression analysis, we investigated the influence of sex and age groups as potential risk factors for several health conditions, including elevated blood pressure (130/85 mm Hg or higher), obesity (BMI 30+ kg/m^2^), elevated glycated haemoglobin (HbA1c) levels (6.5% or higher), and high cholesterol levels. Our findings demonstrated that males exhibited 2.28 times higher odds (95% CI 2.12–2.46) of developing elevated blood pressure than females. Additionally, males had 1.54 times higher odds (95% CI 1.39–1.71) of having elevated HbA1c levels compared to females. Conversely, the odds of obesity were 0.68 times lower (95% CI 0.63–0.73) among males compared to females, as shown in Table 6.

**Table 6:** Factors associated with health risk factors (multi-regression model).

Variable	OR	95% CI	*p* value
Elevated blood pressure – 130/85 mm Hg or higher			
Female (reference)			
Male	2.28	2.12 to 2.46	0.000
18–35 years (reference)			
36+ year	3.58	3.38 to 3.91	0.000
Obese – BMI 30+ kg/m^2^			
Female (reference)			
Male	0.68	0.63 to 0.73	0.000
18–35 years (reference)			
36+ year	2.3	2.15 to 2.46	0.000
Glycated haemoglobin (HbA1c) – 6.5% or higher			
Female (reference)			
Male	1.54	1.39 to 1.71	0.000
18–35 years (reference)			
36+ year	7.65	6.45 to 9.10	0.000
High cholesterol level – cholesterol >6.2 mmol/L			
Female (reference)			
Male	1.61	1.43 to 1.79	0.000
18–35 years (reference)			
36+ year	2.61	2.26 to 2.97	0.000

Furthermore, we observed a significant association between age groups and elevated cholesterol levels. Specifically, individuals aged 36 years and above had 2.61 times higher odds (95% CI 2.26–2.97) of elevated cholesterol levels than younger age groups, as demonstrated in Table 6. The odd ratios showed no difference when calculated in 2017, 2018, and 2019 separately. Consequently, the odds ratios were combined and statistically adjusted across these three years.

## Discussion

The World Health Organization defines chronic disease as an illness of long duration, commonly slow in progression (13). However, chronic disease is a broad category that includes non-communicable diseases (NCDs), such as diabetes, cardiovascular disease, osteoarthritis, chronic obstructive pulmonary disease, cancer, and depression. The diagnosis of (NCDs) can be categorised according to pathophysiology, aetiology, protracted clinical course, comorbidity, symptoms, complications, and treatment. Nevertheless, they all involve a prolonged duration and the absence of a definitive cure (14). The Global Burden of Disease study in 2013 demonstrated a substantial increase in the years lived with disability (YLD) among patients with chronic diseases (15). In Qatar, according to the STEPWise survey conducted by the WHO in 2012, the prevalence of Diabetes among a sample of 2,496 Qataris aged 18 years and over was 16.7% (4).

The current burden of NCDs is related to earlier exposure to accumulative health risks, while the future burden can be attributed to the current exposure to multiple risk factors that could be either non-modifiable, such as age, gender, and genetic vulnerability, or modifiable, such as diet, smoking, and physical activity (16). Additionally, the association between modifiable lifestyle risk factors (alcohol, smoking, body mass index, diet, physical inactivity) and the age to have the first chronic disease was recently established (17).

Therefore, to support people to live healthier and longer, health systems have a key role to play in proactively supporting people to live healthier by taking preventative measures, such as screening programming for early detection of diseases, supporting people to manage long-term chronic conditions, and empowering them to adopt healthier lifestyles (18, 19). As such, the Primary Health Care Corporation in Qatar introduced and implemented the Annual Health Checkup called SMART at its inception in January 2017 for early detection of NCDs and their subsequent risk factors.

This study documents the process and presents the distribution and the pattern of non-communicable diseases and behavioural and metabolic risk factors among the Qatari population who underwent the annual health check assessment appointment among the primary health care corporation target population.

The current analysis found that the uptake of the annual health checkup services increased throughout three years of implementation. This might be attributed to the enhanced communication and social mobilisation activities implemented by the PHCC to engage more of the target population in conducting annual health checkups. According to a rapid review of the NHS Health Check Programme published in 2020, community engagement and invitation phone calls affected increasing the uptake of the annual health screening, especially among ethnic minorities (20). Qatari women exhibited a higher response rate to the annual health checkup invitation throughout the assessment, with twice as many females attending compared to males. In contrast, in the United Kingdom, women continue to be underdiagnosed for cardiovascular diseases, with a 50% lower diagnosis rate compared to men (21). Furthermore, they receive less treatment with medications like cholesterol-lowering and hypertensive drugs (22).

The daily consumption patterns of tobacco remained consistent within our study population throughout the analysis. Qatari males had a higher rate of tobacco consumption compared to females, and this gender difference persisted over the study period. According to data from the epidemiological health assessment in primary care, the tobacco consumption rate was higher among Qatari males than non-Qataris males (23). PHCC has been providing tobacco cessation services via its allocated smoking cessation clinics (24). However, the intensity of the intervention might require an assessment to achieve better outcomes. In a Danish study of health checks, a higher smoking abstinence rate was found in a high-intensity intervention group compared to the usual care (25).

One of the main concerns of our assessed population was the high obesity rate observed. However, in the second year of the annual health checkup implementation, the rates started to decline from 48 to 42.9%. According to the PHCC Health Population Profiling Report for 2022, the prevalence of obesity among non-Qataris in 2021 stood at 36.5% (26). In comparison, obesity rates remained consistently higher among Qataris than among non-Qataris.

The annual health checkup in our analysis demonstrated a key role in detecting pre-diabetic patients among Qataris aged 18+ years. The Glycated Hemoglobin (HbA1c) test was used for the assessment. This test measures the average blood glucose levels over the past two to three months. It indicates long-term blood sugar control. An HbA1c level between 5.7% and 6.4% is typically considered indicative of pre-diabetes (27). Throughout the three years of implementation, pre-diabetic Qataris’ proportion steadily increased. The latter might be attributed to a sedentary lifestyle, unhealthy diets, and the rise in obesity rate (28, 29). Through annual health checkups, pre-diabetic patients were provided with the opportunity to be referred to the wellness services at the PHCC and followed up at the family medicine clinics. The PHCC provides a range of wellness services consisting of healthy lifestyle services. The services include consultations with a multidisciplinary team of professionals and referral to the PHCC wellness centres with a fully equipped gymnasium, a semi-Olympic swimming pool, and group exercise classes (30).

During the lipid profile testing component of the annual health checkup, it was consistently noted that high cholesterol levels (cholesterol >6.2 mmol/L) were prevalent among Qatari males throughout the three years of the assessment. Patients with high cholesterol levels were supported in taking proactive steps, including the referral to PHCC wellness services to manage their cholesterol levels and reduce the risk of developing cardiovascular diseases. The association between elevated levels of cholesterol and cardiovascular diseases is well established. Elevated cholesterol levels, particularly elevated levels of low-density lipoprotein (LDL) cholesterol, are considered a major risk factor for the development of cardiovascular diseases, including coronary artery disease (CAD), heart attacks, and strokes (31).

The strength of this study derived from the ability to include all the eligible population for the annual health checkup and the access that was provided to extract all their test results and social history assessments anonymously. The latter was achieved from the well-designed annual health checkup processes that facilitated the provision of the services across the PHCC operating health centres during the study duration and by the enhanced electronic medical records system enabling data capturing and extraction. Despite the well-defined criteria established for annual health checkups to include all Qataris aged 18 years and above, a potential bias might occur concerning the people with existing medical conditions who might be more likely to undergo the annual health checkup. Additionally, the findings of our analysis might not be applicable to the PHCC Qatari registered population, with the limitation of generalising the findings to the total Qatari population. Another limitation observed was the incomplete social history assessment variable and a few missing test results variables. However, the proportion of missing data was minimal to affect the proportion distribution of risk factors among our studied population.

## Conclusion

The annual health checkup program, implemented between January 1, 2017, and December 31, 2019, prior to the onset of the COVID-19 pandemic in March 2020, proved to be a pivotal tool in identifying and addressing non-communicable diseases (NCDs) and various behavioural and metabolic risk factors among the Qatari population aged 18 years and older. This program served several significant purposes:

### Early detection of NCDs

The annual health checkup program effectively identified individuals at risk of NCDs, such as diabetes, cardiovascular diseases, obesity, and high cholesterol levels. Diagnostic tests and assessments play a crucial role in pinpointing potential health issues at an early stage.

### Behavioural and metabolic risk factors

Beyond disease detection, this program also shed light on behavioural and metabolic risk factors prevalent within the Qatari population. It allowed for the identification of habits like tobacco consumption, sedentary lifestyles, unhealthy diets, and high cholesterol levels, which are critical factors contributing to chronic illnesses.

### Timely intervention

The annual health checkup program did not stop at diagnosis; it provided an avenue for timely intervention. Individuals identified with pre-diabetes, high cholesterol, or other risk factors were referred to the Primary Health Care Corporation’s family medicine clinics and wellness services for comprehensive management and support.

### Enhanced access and data integration

The program’s strength lay in its comprehensive approach, encompassing the entire eligible population. The efficient and well-structured processes, combined with an advanced electronic medical records system, ensured seamless data capture and extraction. This allowed for the thorough analysis of health trends and the development of tailored interventions.

### Public health impact

By identifying and addressing NCDs and risk factors, the annual health checkup program played a crucial role in safeguarding public health. It aimed to reduce the burden of chronic diseases and promote healthier lifestyles among Qataris.

In summary, the annual health checkup program in Qatar was not just a routine assessment; it was a proactive and holistic approach to public health. It facilitated early detection, intervention, and management of NCDs and risk factors, ultimately contributing to the well-being and longevity of the Qatari population. Additionally, it underscored the importance of such preventative measures in healthcare systems worldwide to tackle the growing challenge of chronic diseases.

## Data availability statement

The raw data supporting the conclusions of this article will be made available by the authors, without undue reservation.

## Ethics statement

The studies involving humans were approved by Primary Health Care Corporation Institutional Review Board (IRB) with the following reference number PHCC/DCR/2022/03/09. The studies were conducted in accordance with the local legislation and institutional requirements. The participants consented into conducting the Annual Health Checkups.

## Author contributions

SA-A: Methodology, Supervision, Writing – review & editing. AHB: Formal analysis, Writing – original draft, Writing – review & editing. MM: Project administration, Writing – review & editing. MA-K: Writing – review & editing.
